# Duration and intensity of lactation and maternal risk of subsequent incident coronary artery disease and stroke—a prospective cohort study

**DOI:** 10.1016/j.ajcnut.2025.05.028

**Published:** 2025-05-29

**Authors:** Sophie Hilario Christensen, Julie Aarestrup, Kathleen M Rasmussen, Jennifer L Baker, Dorthe C Pedersen, Lise G Bjerregaard

**Affiliations:** 1 Center for Clinical Research and Prevention, Copenhagen University Hospital—Bispebjerg and Frederiksberg, Copenhagen, Denmark; 2 Department of Nutrition, Exercise and Sports, University of Copenhagen, Frederiksberg, Denmark; 3 Division of Nutritional Sciences, Cornell University, Ithaca, NY, United States

**Keywords:** cohort study, epidemiology, coronary artery disease, lactation, stroke

## Abstract

**Background:**

Although lactation may reduce maternal risk of breast cancer, other potential long-term health benefits of lactation for mothers are largely unknown.

**Objectives:**

We examined whether the durations of predominant and any lactation were associated with maternal risks of coronary artery disease (CAD) and stroke.

**Methods:**

In this prospective cohort study, we followed up 6857 mothers from the Copenhagen Perinatal Cohort who gave birth during 1959–1961 at median age 24 y (IQR, 20–30 y). Durations of predominant and any lactation were assessed at the infant’s 1-y examination. Diagnoses of CAD (*n* = 701 at 45–70 y; *n* = 593 at >70 y) and stroke (*n* = 410 at 45–70 y; *n* = 535 at >70 y) were obtained from national health registers during 1977–2022. Hazard ratios (HRs) and 95% CIs were estimated by Cox regressions without and with adjustment for demographics, metabolic risk during pregnancy, pregnancy complications, and reproductive history.

**Results:**

Durations of predominant and any lactation were inversely associated with risk of CAD, but not with stroke, when using lactation as a continuous variable. In categorical analyses, mothers who lactated for >4 months had 41% (HR: 0.59; 95% CI: 0.46, 0.75) and 34% (HR: 0.66; 95% CI: 0.48, 0.92) lower risk of CAD and stroke, respectively, at ages 45–70 y, compared with mothers who lactated ≤0.5 months. After adjustment for demographic, metabolic, and reproductive risk factors during pregnancy, these associations attenuated (HR: 0.78; 95% CI: 0.60, 1.01 for CAD; HR: 0.90; 95% CI: 0.64, 1.27 for stroke). No associations were observed with CAD or stroke diagnosed after age 70 y.

**Conclusions:**

Limited evidence exists for an association between lactation and maternal risk of stroke. Longer durations of lactation are associated with lower risks of maternal CAD diagnosed before age 70 y. Adjustment for risk factors attenuate the associations, which suggests these factors may partly confound the benefits of lactation on maternal risks of CAD.

## Introduction

Pregnancy is associated with several metabolic changes in the mother, including increased visceral fat, insulin resistance, and circulating lipid concentrations [1]. It has been hypothesized that pregnancy serves as a stress test for cardiovascular disease (CVD), especially when complications such as pre-eclampsia and fetal growth restriction occur [1]. Pregnancy may result in cardiometabolic overload, which can have deleterious long-term effects [1]. Lactation, a component of the reproductive cycle, helps return pregnancy-associated metabolic changes to prepregnancy levels [2]. From this, the reset hypothesis proposes that lactation resets maternal metabolism after pregnancy and thereby reduces later risk of metabolic disease [2]. Some studies show that longer durations of lactation are associated with more favorable changes in insulin, glucose, and lipid homeostasis in mothers several years after delivery [[2], [3], [4]]. Thus, lactation may plausibly confer protection against CVD risks in women even after weaning [5]. Yet, to date, the reset hypothesis has not been adequately tested, and the long-term consequences of lactation on maternal health remain largely unknown [5].

The few studies available on the duration of lactation and incident coronary artery disease (CAD) found inverse associations [[6], [7], [8], [9], [10], [11], [12], [13]], whereas associations with stroke are inconsistent [7,10,11,14,15]. Previous studies were all observational, mostly cohort studies, with sample sizes ranging from 382 to 285,603 women. They are limited by their reliance on recall of lactation behavior obtained many years after it ceased, thus possible effects of misclassification of lactation on the association with CVD outcomes are of great concern. Moreover, studies rarely characterize lactation intensity [e.g., exclusive (only human milk) or any lactation (human milk plus other sources of nutrition)]. Additionally, not all studies accounted for CVD risk factors that occurred before or during pregnancy, which may confound possible associations between lactation and risk of CVD. Adding to these limitations, most studies were conducted in settings where lactation was more common in women with high socioeconomic position, which may have further confounded the associations. Thus, research that tests the independent influence of lactation on risk of CVD is needed to provide evidence for the reset hypothesis.

If lactation reduces maternal cardiovascular disease risk, interventions to promote and prolong lactation may be a viable way to support maternal health. Therefore, we used a Danish cohort with very short recall on lactation intensity and duration to examine whether different durations of predominant and any lactation were associated with maternal risk of incident CAD and stroke, respectively, while accounting for the influence of demographics, metabolic risk during pregnancy, pregnancy complications, and reproductive history. In addition, we performed a negative control outcome analysis in the fathers to assess the potential role of residual or unmeasured confounding.

## Methods

### Cohort

The Copenhagen Perinatal Cohort (CPC) consists of 9125 which, is almost all individuals who were born at the National University Hospital from September 21, 1959, to December 21, 1961 [[16], [17], [18]]. The only births on which data were not collected occurred during the holidays and sick leave of the participating doctors, which were never >1 wk in duration. Hospital admittance was based on area of residence (Copenhagen), but the hospital also preferentially admitted women who previously had pregnancy complications, women who were expected to undergo a complicated delivery, and single women for whom home delivery was impractical. However, most admitted women were residents of Copenhagen and were without pregnancy complications. Women were interviewed regarding social, general medical, and obstetrical history during pregnancy at an antenatal visit at the hospital. The mothers and their children were examined at birth and invited to a follow-up examination of the children at the hospital at 1 y of age [16,17].

### Lactation

The durations of predominant and any breastfeeding (in days, weeks, and/or months) were assessed at the infant’s 1-y examination by a physician who recorded when the mother partly discontinued feeding the infant breast milk (defining the duration of predominant lactation) and when breastfeeding ceased (defining duration of any lactation) [17]. Given the nature of the data, it was not possible to assess the duration of exclusive breastfeeding.

### Covariates

In this study, socioeconomic position of the home at the 1-y examination was scored based on primary income-earner of the household’s occupation, education, how the wage was earned, and the character of the living accommodation. We then divided socioeconomic position into low (0–6 points), middle (7–10 points), and high (11–20 points). In addition to socioeconomic position, we considered the primary income-earner’s education (special needs education, grade school, high school, college), marital status at birth (yes/no), maternal age at delivery (y), prepregnancy BMI (PPBMI, kg/m^2^), smoking during the third trimester (none, <3, 3–10, and >10 cigarettes per day; smoking intensity was not available for trimesters 1 or 2), gestational weight gain (<6, 6–8, 9–10, 11–12, 13–15, and >16 kg), diabetes during pregnancy (yes/no), pre-eclampsia (yes/no), gestational hypertension (yes/no), parity, gestational age (divided into <36 wk, 36–37 wk, and >37 wk due to the original recording of the information), birth weight (kilograms), and sex of the infant [17].

### Follow-up and case ascertainment

Using the unique personal identification number assigned to all Danish residents since 1968, information on vital status, the number of live births, and identification of the father was obtained through linkage to the Danish Civil Registration System [19]. Information on CAD and stroke events among the mothers and fathers was obtained by linkage to the Danish National Patient Register, which was established in 1977 [20]. Age at hospital admission was defined as the age at diagnosis. The outcomes were defined by the International Classification of Diseases (ICD), 8th revision, until 1994 and ICD-10 thereafter (Supplemental Table 1) and included incident CAD (ICD-8: 410.0–414.9; ICD-10: I20–I25) and incident stroke [including ischemic stroke (IS); ICD-8: 431, 433–434, 436; ICD-10: I61, I63, I64]. Participants contributed only the first outcome (stroke prioritized over CAD).

**FIGURE 1:**
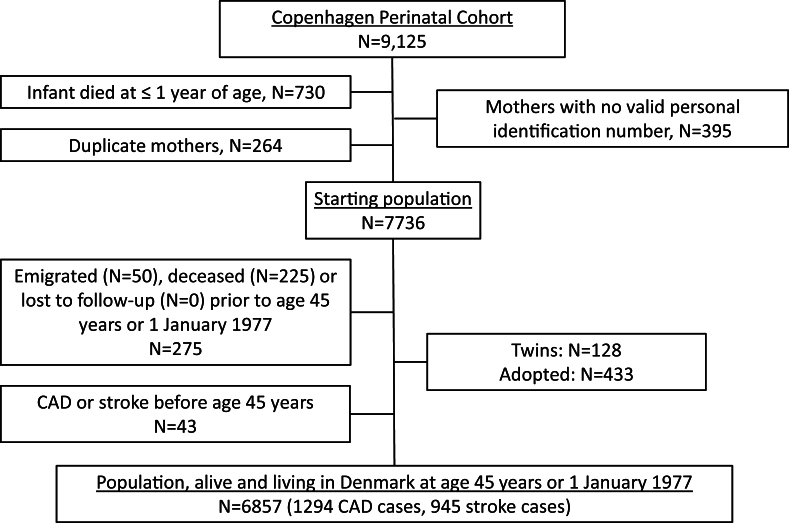
Flow chart of the study population. CAD, coronary artery disease.

Among the mothers of the 9125 children in the CPC, we excluded those with an invalid identification number (Figure 1). If mothers had several children in the CPC, we included only their first participation. To ensure that women no longer had reproductive cycles after start of follow-up, we initiated follow-up at age 45 y or at the inception of the National Patient Register (1 January, 1977), whichever came later. Mothers who emigrated or died before age 45 y or 1 January, 1977, were excluded. We also excluded mothers who had twins or put their child up for adoption. Follow-up of mothers ended at the date of a diagnosis of either CAD or stroke, death, emigration, loss to follow-up, or December 31, 2022; whichever came first. In total, 6857 mothers were included in the analyses, of whom 6041 had information on the duration of lactation.

According to Danish law, ethical approval is not required for purely questionnaire and register-based studies unless they involve human biological material. Thus, this study is exempt for ethical approval. Instead, it is covered by approval from the Danish Data Protection Agency (P-2019-832) that supervises data security and proper use of data in research where individuals are not contacted.

### Statistical analysis

Key characteristics (percentages or median and IQR) are presented for the participants overall and by categories of duration of lactation. The median duration of follow-up was calculated using the reverse Kaplan–Meier method [21]. Hazard ratios (HRs) and 95% CIs for the associations between the duration of lactation and CAD and stroke, respectively, were estimated using Cox proportional hazards regression models. In addition, we analyzed IS separately.

Age was used as the underlying timescale. We treated the durations of predominant and any lactation as continuous variables (in months). Moreover, any lactation was divided into 5 groups (≤0.5, >0.5–1, >1–2, >2–4, and >4 mo) reflecting the duration of lactation in this population. The reference group was women who lactated ≤0.5 mo.

Potential confounding factors were selected a priori based on established associations and/or plausible biological relationships. Directed acyclic graphs were used to identify which factors to adjust for based on the assumed associations (Supplemental Figures 1 and 2). Three models were used to investigate the impact of stepwise adjustment for *1*) social and prepregnancy-related factors [socioeconomic position, maternal age at delivery (years), and PPBMI], *2*) pregnancy metabolic risk factors (smoking during the third trimester, diabetes, pre-eclampsia and/or hypertension during pregnancy), and *3*) a reproductive history and birth complications (parity before the index birth, gestational age and birth weight of the child).

The proportion of missing data was <5% for most putative confounders but was 10% for PPBMI, 17% for socioeconomic position, and 19% for gestational age. Thus, multiple imputations of duration of lactation and the potential confounders were performed using chained equations with 40 imputations as implemented in Stata through the “mi” command based on all covariates, lactation, and the outcomes [22].

Potential effect modification of the association between lactation and risk of CVD outcomes by socioeconomic position and the number of live births given after the index birth, respectively, was examined by introducing interaction terms in the Cox model and examining their significance using Wald tests. We found no indication of interactions with these 2 factors (all *P* values ≥ 0.48). The proportional hazards assumption underlying the Cox model was investigated by including an interaction by categories of age at risk (divided by the overall median of age at diagnosis; 45–70 or >70 y) using the likelihood ratio test. As these tests were significant for most outcomes, all analyses were performed by separating the follow-up time at ages 45–70 y and >70 y.

To assess potential residual or unmeasured confounding that may create a spurious association between lactation and CVD risk in the mother, risk of CVD outcomes in the fathers was used as a negative control outcome [23] and analyzed in a subsample with identification of the father (Supplemental Figure 3). As lactation does not have a causal effect on paternal morbidity, an association between lactation and paternal CVD would indicate bias due to for instance confounding from sociodemographic factors and the difference in paternal and maternal associations likely reflects the association due to lactation.

In a sensitivity analysis, we assessed whether exposure to more reproductive cycles (for which we do not have information on lactation) than the one related to the index pregnancy influences the associations. We did this by investigating the association between lactation and risk of CVD outcomes in mothers with 1 child. In these women we had information on the lifetime history of lactation. All analyses were conducted in Stata version 17.0 [22].

## Results

Approximately 24% of mothers lactated for ≤0.5 mo, 15% for >0.5–1 mo, 20% for >1–2 mo, 22% for >2–4 mo, and 21% for >4 mo. Mothers who lactated for ≤0.5 mo, compared with mothers who lactated longer, were more often from homes of lower socioeconomic position, more often having prepregnancy overweight or obesity, and more often had been diagnosed with diabetes during pregnancy (Table 1) [17]. The median follow-up time was 35.4 y (188,500 person-years in total). During this period, 1294 (19%) mothers were diagnosed with CAD, and 945 (14%) were diagnosed with stroke, of which 840 (12%) were IS. Of these, 54.2%, 43.4%, and 42.6%, respectively, occurred before age 70 y.

**TABLE 1:** Characteristics of the study population by duration of lactation^1^.

Characteristic	Data available (n)	Total	Duration of any lactation (mo)				
			≤0.5	>0.5–1	>1–2	>2–4	>4
N in group	6041		1363	885	1185	1352	1256
Socioeconomic position^2^	5701						
Low		1308 (22.9)	351 (29.5)	228 (27.9)	263 (24.3)	256 (20.4)	155 (13.0)
Medium		2425 (42.5)	541 (45.5)	390 (47.7)	476 (44.0)	534 (42.6)	424 (35.6)
High		1968 (34.5)	298 (25.0)	199 (24.4)	342 (31.6)	464 (37.0)	613 (51.4)
Primary income-earner of the household’s education	5643						
Special need education		70 (1.2)	21 (1.8)	14 (1.8)	11 (1.0)	9 (0.7)	6 (0.5)
Grade school		3710 (65.7)	854 (74.6)	608 (76.1)	747 (69.5)	795 (64.6)	606 (52.2)
High school		1238 (21.9)	214 (18.7)	136 (17.0)	216 (20.1)	301 (24.5)	309 (26.6)
College		625 (11.1)	56 (4.9)	41 (5.1)	101 (9.4)	125 (10.2)	241 (20.7)
Marital status: married	6776	4343 (64.1)	828 (61.8)	508 (58.0)	696 (59.5)	869 (65.2)	997 (79.8)
Age at delivery (y), median (IQR)	6856	24 (20–30)	24 (20–31)	23 (19–28)	23 (19–29)	24 (20–30)	27 (22–32)
PPBMI (kg/m^2^) median (IQR)	6155	21.4 (19.8–23.1)	21.5 (19.9–23.6)	21.4 (19.7–23.1)	21.2 (19.7–22.9)	21.2 (19.8–22.9)	21.4 (19.8–23.0)
PPBMI (kg/m^2^)	6155						
<18.5		538 (8.7)	122 (10.2)	78 (9.9)	106 (9.7)	106 (8.7)	66 (5.7)
≥18.5 and <25		4943 (80.3)	890 (74.3)	633 (80.4)	887 (81.5)	989 (80.8)	975 (84.4)
≥25 and <30		583 (9.5)	153 (12.8)	64 (8.1)	87 (8.0)	115(9.4)	102 (8.8)
≥30		91 (1.5)	33 (2.8)	12 (1.5)	9 (0.8)	14 (1.1)	12 (1.0)
Smoking during the third trimester (cigarettes/d)	6759						
None		3337 (49.4)	607 (45.3)	388 (44.3)	542 (46.2)	643 (48.2)	784 (63.4)
<3 daily		486 (7.2)	87 (6.5)	65 (7.4)	90 (7.7)	95 (7.1)	86 (7.0)
3–10 daily		2005 (29.7)	406 (30.3)	271 (30.9)	356 (30.4)	434 (32.5)	288 (23.3)
>10 daily		931 (13.8)	239 (17.8)	152 (17.4)	184 (15.7)	163 (12.2)	79 (6.4)
Gestational weight gain (kg)	4085						
<6		288 (7.1)	70 (9.1)	37 (7.0)	44 (6.0)	51 (6.1)	47 (6.2)
6–8		662 (16.2)	152 (19.8)	90 (17.1)	110 (14.9)	134 (16.1)	95 (12.5)
9–10		728 (17.8)	140 (18.2)	97 (18.4)	144 (19.5)	155 (18.6)	122 (16.1)
11–12		741 (18.1)	115 (15.0)	84 (15.9)	125 (16.9)	156 (18.8)	166 (21.8)
13–15		866 (21.29	134 (17.4)	103 (19.5)	167 (22.6)	170 (20.4)	188 (24.7)
≥16		800 (19.6)	157 (20.4)	116 (22.0)	149 (20.2)	166 (20.0)	142 (18.7)
Maternal diabetes during pregnancy: yes	6856	61 (0.99)	24 (1.8)	6 (0.7)	5 (0.4)	—3	—3
Pre-eclampsia: yes	6823	277 (4.1)	61 (4.5)	36 (4.1)	40 (3.4)	54 (4.0)	61 (4.9)
Gestational hypertension: yes	6780	1258 (18.6)	235 (17.6)	149 (17.1)	216 (18.4)	257 (19.1)	268 (21.5)
Parity, median (IQR)	6857	1.0 (1.0–2.0)	1 (1–2)	1 (1–2)	1 (1–2)	1 (1–2)	1 (1–2)
Gestational age	5563						
<36 wk		388 (7.0)	138 (12.7)	37 (5.3)	60 (6.3)	68 (6.2)	44 (4.2)
36–37 wk		594 (10.7)	149 (13.7)	70 (10.0)	80 (8.4)	118 (10.8)	104 (9.9)
>37 wk		4581 (82.39	803 (73.7)	595 (84.8)	815 (85.3)	908 (83.0)	905 (85.9)
Birth weight (kg), median (IQR)	6733	3.25 (2.9–3.6)	3.15 (2.65. 3.55)	3.25 (2.95. 3.6)	3.25 (2.95. 3.6)	3.3 (2.95. 3.6)	3.35 (3. 3.7)
Sex of the child: boys	6857	3477 (50.7)	716 (52.5)	438 (49.5)	608 (51.3)	687 (50.8)	628 (50.0)
Duration of predominant lactation (mo)	5980	1.4 (0.5–3.0)	0.2 (0.2–0.2)	0.8 (0.7–1.0)	1.5 (1.0–2.0)	3.0 (2.0–3.0)	4.5 (3.0–6.0)

Abbreviation: PPBMI, prepregnancy BMI.

1 The table provides the number of participants with information available and *n* and column percentages (%) within a given category of the characteristics or median and IQR.

2 Points for socioeconomic position were given according to occupation of the primary income-earner of the household, the way in which the primary income-earner earn his/her wages, the training of the primary income-earner and the character of the living accommodation (0–5 point per indicator) [17]. Socioeconomic position was divided into low (0–6 points), medium (7–10 points), or high (11–20 points).

3 These numbers cannot be shown according to data protection rules set by the national statistics service (Denmark Statistics) due to low numbers in one of the categories.

In the unadjusted analyses, the durations of predominant and any lactation were associated with a 7% reduced risk of CAD diagnosed between ages 45 and 70 y (HR: 0.93; 95% CI: 0.89, 0.98; HR: 0.93; 95% CI: 0.90, 0.96, per each additional month of predominant and any lactation). The associations attenuated after adjustment for social, metabolic, and reproductive factors, but the association between the duration of any lactation and CAD was significant even in the fully adjusted analysis (HR: 0.97; 95% CI: 0.93, 1.00 per each additional month of lactation; *P* = 0.04). No associations were found between the durations of predominant or any lactation and risk of stroke or IS either before or after adjustment (Table 2).

**TABLE 2:** Associations between duration of predominant and any lactation and risk of CAD and stroke at ages 45–70 y in 6857 mothers and at ages >70 years in 4495 mothers^1^.

Age at risk (y)	Exposure	CAD, HR (95% CI), *n* = 701		Stroke, HR (95% CI), *n* = 410		IS, HR (95% CI), *n* = 358	
		Unadjusted	Fully adjusted^2^	Unadjusted	Fully adjusted^2^	Unadjusted	Fully adjusted^2^
45–70	Predominant lactation						
	Per mo	0.93 (0.89, 0.98)	0.97 (0.93, 1.02)	0.98 (0.92, 1.04)	1.02 (0.96, 1.08)	1.00 (0.95, 1.06)	1.05 (0.99, 1.12)
	Any lactation						
	Per mo	0.93 (0.90, 0.96)	0.97 (0.93, 1.00)^3^	0.97 (0.94, 1.01)	1.01 (0.97, 1.05)	0.99 (0.95, 1.03)	1.03 (0.99, 1.07)
>70	Predominant lactation						
	Per mo	1.00 (0.97, 1.03)	1.00 (0.95, 1.05)	1.01 (0.96, 1.06)	1.02 (0.97, 1.07)	1.00 (0.96, 1.06)	1.01 (0.96, 1.06)
	Any lactation						
	Per mo	0.98 (0.94, 1.03)	1.01 (0.98, 1.04)	0.98 (0.95, 1.01)	0.99 (0.96, 1.02)	0.98 (0.95, 1.01)	0.98 (0.95, 1.02)

Abbreviations: CAD, coronary artery disease; HR, hazard ratio; IS, ischemic stroke.

1 HRs and 95% CIs were estimated by Cox proportional hazards regression analyses.

2 The estimates were adjusted for socioeconomic positions (low, medium, and high), maternal age (years), prepregnancy BMI (kg/m^2^), maternal smoking (none, <3, 3–10, >10 cigarettes per day) and diabetes during pregnancy (yes/no), pre-eclampsia (yes/no), gestational hypertension (yes/no), parity, gestational age (<36, 36–37, and >37 wk), and birth weight (kg).

3 *P* = 0.040.

**FIGURE 2:**
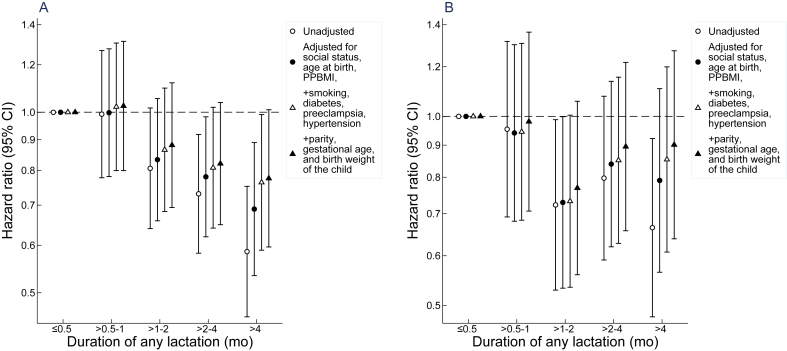
Associations between the duration of any lactation and risk of coronary artery disease (A) and stroke (B) at ages 45–70 y in mothers. The shown hazard ratios and 95% CIs were estimated by Cox proportional hazards regression analyses. The estimates are adjusted for socioeconomic position, maternal age, prepregnancy BMI (PPBMI), maternal smoking and diabetes during pregnancy, pre-eclampsia, gestational hypertension, parity, gestational age, and birth weight. The error bars represent the 95% CIs.

When dividing the duration of any lactation into categories, mothers who lactated >2–4 mo had an unadjusted HR of 0.73 (95% CI: 0.58, 0.92), and mothers who lactated >4 mo had an HR of 0.59 (95% CI: 0.46, 0.75) for risk of CAD diagnosed at ages 45–70 ys compared with mothers who lactated ≤0.5 mo (Figure 2; Supplemental Table 2). The corresponding unadjusted HRs for stroke were 0.80 (95% CI: 0.59, 1.08) and 0.66 (95% CI: 0.48, 0.92). The results for CAD and stroke attenuated gradually after the stepwise adjustment for especially social and metabolic factors, but only slightly after further adjustment for reproductive factors (Figure 2). In the fully adjusted model, mothers who lactated for >4 mo had 22% (HR: 0.78; 95% CI: 0.60, 1.01) reduced risk of CAD and 10% reduced risk of stroke (HR: 0.90; 95% CI: 0.64, 1.27), respectively. In the unadjusted model, the HRs for IS were 0.70 (95% CI: 0.49, 0.98), 0.87 (95% CI: 0.63, 1.20), and 0.71 (95% CI: 0.51, 1.00) associated with lactation for >1–2, >2–4, and >4 mo, respectively. After adjustment, the estimates shifted toward the null with CIs overlapping the null (Supplemental Table 2).

We found no associations between predominant or any lactation and any of the outcomes diagnosed >70 y of age. The estimates for CAD were close to 1 in all analyses (Table 2; Supplemental Figure 4; Supplemental Table 3).

Using CVD outcomes in the fathers as negative controls, the duration of lactation was not associated with any of the outcomes (Supplemental Table 4). In the sensitivity analyses conducted on 1285 women who only had 1 child, no statistically significant results were found when using duration of lactation as a continuous variable, and the estimates had wide CIs (Supplemental Table 5). In the categorical analysis, we found inverse associations between the duration of lactation and risks of CAD and stroke; however, these associations were mostly nonsignificant (Supplemental Table 5).

## Discussion

In this cohort, we found an inverse association between the duration of any lactation and maternal risk of CAD at ages 45–70 y. The associations attenuated after adjustment for well-known risk factors, including social background and pregnancy-related factors. This indicates that these factors partly explain the associations. Moreover, the benefit of lactation was no longer evident after the age of 70 y. We found limited evidence of an association between lactation and maternal risk of stroke. The duration of lactation was not associated with paternal CVD outcomes in the negative control analysis, suggesting that there is limited unmeasured or residual confounding of the observed inverse association between lactation and CAD risk in the mother.

When analyzing the duration of lactation continuously, we found that each additional month of any lactation was associated with a 3% lower risk of CAD at ages 45–70 y. In comparison, a meta-analysis from 2022 based on 6 large studies reported a slightly weaker association [24]. When assuming a linear trend for the lifetime duration of lactation, each additional year of lactation was associated with a 11% (0%–20%) reduced risk of CAD, corresponding to ∼1% per additional month [24]. Differences in the age at inclusion may explain the contrasting results, as the mean age at study entry and thus age at reporting lactation was 51.3 y in the meta-analysis [24]. We found no significant association with stroke when analyzing the duration of predominant and any lactation continuously. Similarly, the meta-analysis reported a nonsignificant HR of 0.91 (95% CI: 0.78, 1.06) for stroke per each additional year [24].

It may be speculated that the duration of predominant lactation would have a stronger association with CVD outcomes than the duration of any lactation. This is because any lactation combines all intensities of lactation, whereas predominant lactation has a systematically higher intensity than this. However, the associations were of similar strength for predominant and any lactation. Thus, it may be speculated that the associations of lactation behavior with disease risk are independent of lactation intensity. Importantly, the durations of any and predominant lactation were relatively similar in our study. Most other studies did not have information on the intensity of lactation. Only 1 other Danish study had this information available. However, a direct comparison is challenging as the intensity of lactation was only examined in combination with the duration of partial lactation (the duration of any lactation minus the duration of full lactation) and not compared with no lactation [11]. Moreover, the women in the other study were born decades after our study population and were younger at follow-up, further limiting a comparison.

A strength of our study is the long follow-up period, which allowed us to investigate the associations between the duration of lactation and CAD and stroke in both middle and older ages. We found no associations with CVD diagnosed after the age of 70 y, indicating that the protective association of lactation with CVD wanes over time. This finding may be explained by the longer time period from lactation to the outcome during which other CVD risk factors become more prevalent and more important. These findings are in accord with those from other studies [3,6,25]. For example, a study using data from the Women’s Health Initiative found that a longer duration of any lactation was associated with reduced risk of CVD in women aged 50–59 y at enrollment into the cohort, whereas the associations were weaker at ages 60–69 y and nonexistent among women aged 70–79 y [3]. Similarly, a Norwegian study found an association between lactation and CVD mortality only in women younger than 65 y at baseline [25]. Moreover, in a study within the Nurses’ Health Study, there was an association between lactation and myocardial infarction in women who had given birth in the previous 30 y, but not in women whose last birth was >30 y ago [6]. In contrast, a recent meta-analyses of lactation and maternal risk of CAD and CVD reported that associations did not differ according to mean age at baseline from 40 to 60 y or according to median follow-up time (≤20 y) [24]. Taken together, our results and the published evidence suggest that the cardiovascular benefits of lactation decrease with time since lactation, but more studies are needed to verify these findings.

Several previous studies were conducted in settings where women of higher socioeconomic position lactated for longer durations [3,9,15,25]. Thus, the duration of lactation might be a marker of a high socioeconomic position and related health-promoting behaviors rather than having a biological effect on risk of CVD morbidity. Supporting this, other studies showed that associations between lactation and CVD outcomes diminished after adjustment for other CVD risk factors [3,9]. In contrast, minimal effects of adjusting for socioeconomic indicators have also been reported in some studies [6,8,10,15,25], suggesting that confounding from socioeconomic position may be limited. We found that the associations attenuated after adjustment for socioeconomic position reflecting that mothers who lactated longer were more often from homes of higher socioeconomic position. Nevertheless, the lack of association between duration of lactation and CVD outcomes in the fathers provides evidence that there is limited unmeasured confounding or residual confounding by socioeconomic factors. Although we cannot preclude this completely, since socioeconomic position may affect maternal risk of CVD more than that for paternal risk of CVD, our results support that the observed protective benefit of lactation may not entirely be explained by socioeconomic position.

Possible pathophysiologic mechanisms for our findings include hormonal responses related to reproduction and lactation. For instance, oxytocin, which is released during lactation, improves glucose tolerance and lowers blood pressure [26]. Lactation may mobilize accumulated fat stores after delivery [2] and may, even after adjustment for sociodemographic factors, facilitate a faster resetting of pregnancy-related cardiometabolic changes. These include hypertension, diabetes, and hyperlipidemia [3], which are precursors of CVD outcomes. Moreover, lactation has been shown to decrease stress and concentrations of proinflammatory markers [27,28] also after controlling for sociodemographic variables [27]. Together, these beneficial effects may reduce risk of CVD outcomes, especially if they recur across several reproductive cycles.

Compared with other studies on the duration of lactation and maternal risk of CVD outcomes, one of the major strengths of our study is that the data on the duration of lactation were collected shortly after cessation, limiting the potential for misclassification associated with recall of duration of lactation. Except for 1 study [11], all other studies in this area relied on self-reported information on lactation collected up to decades after cessation, thus the accuracy of the data are of concern. Women more often overreport rather than underreport duration of lactation [29]. This may cause the associations to be underestimated and, thus, could potentially explain the weaker findings in the meta-analysis [24]. We were able to adjust for a wide range of potential confounding factors, including PPBMI and socioeconomic position, and we analyzed a negative control outcome. Most other studies did not adjust for CVD risk factors during pregnancy.

Another strength of this study is the nearly complete follow-up through national registers. Attrition in our study resulted mainly from infant mortality and missing identification numbers. Mothers with a complicated birth may be overrepresented in the CPC. Thus, some selection into the cohort may have occurred. Pregnancy complications are associated with increased risks of CVD outcomes [30,31] and shorter duration of lactation, hence lactation may be a marker of CVD factors occurring during pregnancy, which may result in reverse causation. However, we were able to adjust for pregnancy-related factors. Thus, we find it unlikely that our analysis overestimates the effect of lactation for this reason.

Unlike most other studies that used self-reported information on the outcome, we used objectively assessed information on CAD and stroke. The coverage of the Danish National Patient Register is high, with virtually every event recorded [20]. Moreover, the positive predictive values for myocardial infarction and angina pectoris are 88%–97% [32] and 72%–100%, respectively, for IS, unspecified stroke, and overall stroke. Thus, risk of potential misclassification of the outcome is low.

Our study also has limitations. Although lactation was encouraged during the study period [33], the duration of lactation was relatively short compared with current recommendation and recommendations at the time the cohort was exclusive breastfeeding for 5–6 mo [34]. Nonetheless, the duration of any lactation at >4 mo (20%) was in the range of observed proportions among infants born in (32%) and out (18%) of wedlock in the Copenhagen Municipality in the years 1958–1962 [35]. Moreover, information on the duration of lactation came from only 1 pregnancy and was used as an indicator of the average duration of lactation for all the individuals’ subsequent births as data on the behavior were not available. However, Danish studies on children born in 1977–1979 and in 1986 reported that mothers who had breastfed an older child usually breastfed the next child for a similar time [36,37]. Additionally, we found similar results in the sensitivity analyses in mothers with 1 child, although with less statistical power. Further studies are needed to investigate whether the findings are generalizable to other and more contemporary populations in which durations of lactation are longer.

In conclusion, we found that longer lactation following 1 singleton pregnancy was associated with a lower risk of maternal CAD. This association was no longer evident after 70 y of age. We found limited evidence of an association between lactation and maternal risk of stroke. The adjustment for demographics, metabolic risk during pregnancy, and reproductive history weakened the associations, suggesting that these factors may partly explain the CVD benefits of lactation on maternal risk of CAD and stroke. Nonetheless, our findings indicate that longer durations of lactation are associated with reduced risks of later CAD.

## Author contributions

The authors’ responsibilities were as follows – LGB, SHC: designed the research; JLB, LGB: provided the databases; LGB: conducted the analyses; DCP, JA, JLB, KMR: contributed to the specification of the analyses; LGB, SHC: wrote the manuscript; LGB: had primary responsibility for the final content; and all authors: critically read and edited the manuscript and approved the final manuscript.

## Data availability

The data used in this study are based on a combination of data with personal identification numbers from the Copenhagen Perinatal Cohort, hosted by the Center for Clinical Research and Prevention, and data from national health registers. According to Danish law, this information cannot be made publicly available. Access to the subset of data included in this study requires the submission of a project application to the corresponding author, Lise G. Bjerregaard, and approval by the data steering committee.

## Funding

This work was supported by grants awarded to LGB from the Danish Heart Foundation (21-R150-A9850-22196), Helene og Georg Jensens samt Ethel Merethe og Christian Pontoppidans Fond, and the Novo Nordisk Foundation (NNF21OC0071349).

## Conflict of interest

KMR is a member of the Editorial Board of the *American Journal of Clinical Nutrition* and had no role in the Journal’s evaluation of the manuscript. The other authors report no conflicts of interest.
