# Supplementary material for: Duration and intensity of lactation and maternal risk of subsequent incident coronary artery disease and stroke—a prospective cohort study
Source: Am J Clin Nutr. 2025 May 29;122(2):433–40. doi: 10.1016/j.ajcnut.2025.05.028 (PMC12489343; doi:10.1016/j.ajcnut.2025.05.028)

**Supplementary information**

**Supplementary Table 1. ICD-codes for incident coronary heart disease and stroke.** The diagnoses were coded according to the ICD-8 until the end of 1993 and according to the ICD-10 thereafter.

|  | **CHD** | **Total Stroke** | **IS** |
| --- | --- | --- | --- |
| ICD-8 | 410: Acute cardiac infarction | 431: Cerebral haemorrhage |  |
|  | 411: Other acute and subacute forms of CHD | 433: Cerebral thrombosis | 433: Cerebral thrombosis |
|  | 412: Old cardiac infarction or chronic CHD | 434: Cerebral embolism | 434: Cerebral embolism |
|  | 413: Angina pectoris | 436: Acute but ill-defined cerebrovascular disease^1^ | 436: Acute but ill-defined cerebrovascular disease |
|  | 414: Asymptomatic CHD |  |  |
| ICD-10 | I20: Angina pectoris | I61: Intracerebral haemorrhage |  |
|  | I21: Acute cardiac infarction | I63: Cerebral infarction | I63: Cerebral infarction |
|  | I22: Reinfarction (within 4 weeks) | I64: Stroke, not specified as haemorrhage or infarction | I64: Stroke, not specified as haemorrhage or infarction |
|  | I23: Complications due to acute cardiac infarction |  |  |
|  | I24: Other acute forms of CHD |  |  |
|  | I25: Chronic CHD |  |  |

^1^ Incident ischemic stroke includes unspecified stroke (ICD-8: 436, ICD-10: I64) because >60% of the unclassified strokes in Denmark are ischemic (38).

CHD, coronary heart disease; ICD, International Classification of Diseases; IS, Ischemic stroke.

**Supplementary Table 2.** Associations between duration of any lactation and risk of CHD and stroke at ages 45 to 70 years in 6,857 mothers.

|  | **CHD (HR, 95%CI)** | | **Stroke (HR, 95%CI)** | | **IS (HR, 95%CI)** | |
| --- | --- | --- | --- | --- | --- | --- |
| No. of cases | 701 | | 410 | | 358 | |
| Exposure | Unadjusted | Fully adjusted^a^ | Unadjusted | Fully adjusted^a^ | Unadjusted | Fully adjusted^a^ |
| Any lactation (month) |  |  |  |  |  |  |
| ≤0.5 | 1.00 (Reference) | 1.00 (Reference) | 1.00 (Reference) | 1.00 (Reference) | 1.00 (Reference) | 1.00 (Reference) |
| >0.5-1 | 0.99 (0.78-1.27) | 1.02 (0.80-1.31) | 0.95 (0.69-1.32) | 0.98 (0.71-1.36) | 0.92 (0.65-1.31) | 0.95 (0.66-1.36) |
| >1-2 | 0.81 (0.64-1.02) | 0.88 (0.69-1.12) | 0.72 (0.53-0.99) | 0.77 (0.56-1.06) | 0.70 (0.49-0.98) | 0.74 (0.52-1.06) |
| >2-4 | 0.73 (0.58-0.92) | 0.82 (0.65-1.04) | 0.80 (0.59-1.08) | 0.90 (0.66-1.22) | 0.87 (0.63-1.20) | 0.98 (0.71-1.36) |
| >4 | 0.59 (0.46-0.75) | 0.78 (0.60-1.01) | 0.66 (0.48-0.92) | 0.90 (0.64-1.27) | 0.71 (0.51-1.00)^b^ | 0.97 (0.68-1.39) |

^a^ The estimates are adjusted for socioeconomic position, maternal age, pre-pregnancy body mass index, maternal smoking and diabetes during pregnancy, preeclampsia, gestational hypertension, parity, gestational age, and birth weight.

^b^ P=0.051.

CHD, coronary heart disease; CI, confidence interval; HR, hazard ratio; IS, ischemic stroke.

**Supplementary Table 3.** Associations between duration of any lactation and risk of CHD and stroke at ages >70 years in 4,495 mothers.

|  | **CHD (HR, 95%CI)** | | **Stroke (HR, 95%CI)** | | **IS (HR, 95%CI)** | |
| --- | --- | --- | --- | --- | --- | --- |
| No. of cases | 593 | | 535 | | 482 | |
| Exposure | Unadjusted | Fully adjusted^a^ | Unadjusted | Fully adjusted^a^ | Unadjusted | Fully adjusted^a^ |
| Any lactation |  |  |  |  |  |  |
| ≤0.5 | 1.00 (Reference) | 1.00 (Reference) | 1.00 (Reference) | 1.00 (Reference) | 1.00 (Reference) | 1.00 (Reference) |
| >0.5-1 | 1.01 (0.75-1.37) | 1.04 (0.77-1.41) | 0.71 (0.50-0.99) | 0.72 (0.51-1.01) | 0.70 (0.49-0.99) | 0.70 (0.49-1.00)^b^ |
| >1-2 | 1.01 (0.77-1.33) | 1.07 (0.81-1.41) | 0.91 (0.69-1.19) | 0.96 (0.73-1.27) | 0.91 (0.68-1.23) | 0.95 (0.71-1.29) |
| >2-4 | 1.01 (0.78-1.32) | 1.07 (0.82-1.39) | 0.88 (0.67-1.15) | 0.93 (0.70-1.23) | 0.92 (0.69-1.22) | 0.95 (0.71-1.27) |
| >4 | 1.03 (0.80-1.32) | 1.11 (0.86-1.44) | 0.81 (0.62-1.06) | 0.86 (0.65-1.13) | 0.82 (0.62-1.09) | 0.85 (0.64-1.14) |

^a^ The estimates are adjusted for socioeconomic position, maternal age, pre-pregnancy body mass index, maternal smoking and diabetes during pregnancy, preeclampsia, gestational hypertension, parity, gestational age, and birth weight.

^b^ p=0.051.

CHD, coronary heart disease; CI, confidence interval; HR, hazard ratio; IS, ischemic stroke.

**Supplementary Table 4.** Associations between duration of predominant and any lactation and risk of CHD and stroke at ages 45 to 70 years in 6,057 fathers.

|  | **CHD**  **(HR, 95%CI)** | **Stroke**  **(HR, 95%CI)** | **IS**  **(HR, 95%CI)** |
| --- | --- | --- | --- |
| No. of cases | 1079 | 448 | 393 |
| Exposure | Fully adjusted^a^ | Fully adjusted^a^ | Fully adjusted^a^ |
| Predominant lactation |  |  |  |
| Per month | 0.99 (0.95-1.02) | 1.06 (1.00-1.11) | 1.07 (1.01-1.13) |
| Any lactation |  |  |  |
| Per month | 0.99 (0.97-1.02) | 1.01 (0.97-1.04) | 1.01 (0.98-1.05) |
| ≤0.5 | 1.00 (Reference) | 1.00 (Reference) | 1.00 (Reference) |
| >0.5-1 | 1.09 (0.89-1.34) | 0.90 (0.64-1.27) | 0.81 (0.56-1.18) |
| >1-2 | 0.99 (0.81-1.20) | 1.01 (0.75-1.36) | 0.93 (0.67-1.29) |
| >2-4 | 0.97 (0.80-1.18) | 1.14 (0.85-1.52) | 1.11 (0.82-1.51) |
| >4 | 0.95 (0.77-1.17) | 1.04 (0.77-1.42) | 1.02 (0.74-1.42) |

^a^ The estimates are adjusted for socioeconomic position, maternal smoking during pregnancy, and parity.

CHD, coronary heart disease; CI, confidence interval; HR, hazard ratio; IS, ischemic stroke.

**Supplementary Table 5.** Associations between duration of predominant and any lactation and risk of CHD and stroke at ages 45 to 70 years in 1,285 mothers with one child.

|  | **CHD (HR, 95%CI)** | | **Stroke (HR, 95%CI)** | | **IS (HR, 95%CI)** | |
| --- | --- | --- | --- | --- | --- | --- |
| No. of cases | 112 | | 69 | | 59 | |
| Exposure | Unadjusted | Fully adjusted^a^ | Unadjusted | Fully adjusted^a^ | Unadjusted | Fully adjusted^a^ |
| Predominant lactation |  |  |  |  |  |  |
| Per month | 1.00 (0.90-1.11) | 1.04 (0.93-1.16) | 0.93 (0.79-1.10) | 0.97 (0.82-1.14) | 0.97 (0.82-1.13) | 1.00 (0.85-1.18) |
| Any lactation |  |  |  |  |  |  |
| Per month | 0.98 (0.91-1.05) | 1.00 (0.93-1.07) | 0.93 (0.84-1.04) | 0.96 (0.86-1.08) | 0.95 (0.85-1.06) | 0.98 (0.88-1.10) |
| ≤0.5 | 1.00 (Reference) | 1.00 (Reference) | 1.00 (Reference) | 1.00 (Reference) | 1.00 (Reference) | 1.00 (Reference) |
| >0.5-1 | 0.82 (0.46-1.46) | 0.83 (0.45-1.50) | 0.64 (0.30-1.37) | 0.63 (0.29-1.38) | 0.78 (0.35-1.74) | 0.79 (0.35-1.81) |
| >1-2 | 0.57 (0.30-1.05) | 0.58 (0.31-1.11) | 0.29 (0.12-0.75) | 0.30 (0.12-0.78) | 0.32 (0.11-0.92) | 0.35 (0.12-1.00)^b^ |
| >2-4 | 0.59 (0.33-1.05) | 0.71 (0.39-1.29) | 0.58 (0.29-1.17) | 0.66 (0.32-1.36) | 0.68 (0.33-1.40) | 0.78 (0.37-1.64) |
| >4 | 0.65 (0.36-1.16) | 0.78 (0.42-1.45) | 0.40 (0.18-0.92) | 0.52 (0.22-1.22) | 0.49 (0.21-1.18) | 0.65 (0.26-1.61) |

^a^ The estimates are adjusted for socioeconomic position, maternal age, pre-pregnancy body mass index, maternal smoking and diabetes during pregnancy, preeclampsia, gestational hypertension, parity, gestational age, and birth weight.

^b^ P=0.050009.

CHD, coronary heart disease; CI, confidence interval; HR, hazard ratio; IS, ischemic stroke.

**Supplementary Figure 1.** Conceptual framework, unadjusted (pink arrows indicate bias). CHD, coronary heart disease; Gest, Gestional; GWG, Gestational weight gain; Mat, Maternal; Pre-pregBMI, pre-pregnancy body mass index; SES: Socioeconomic position.


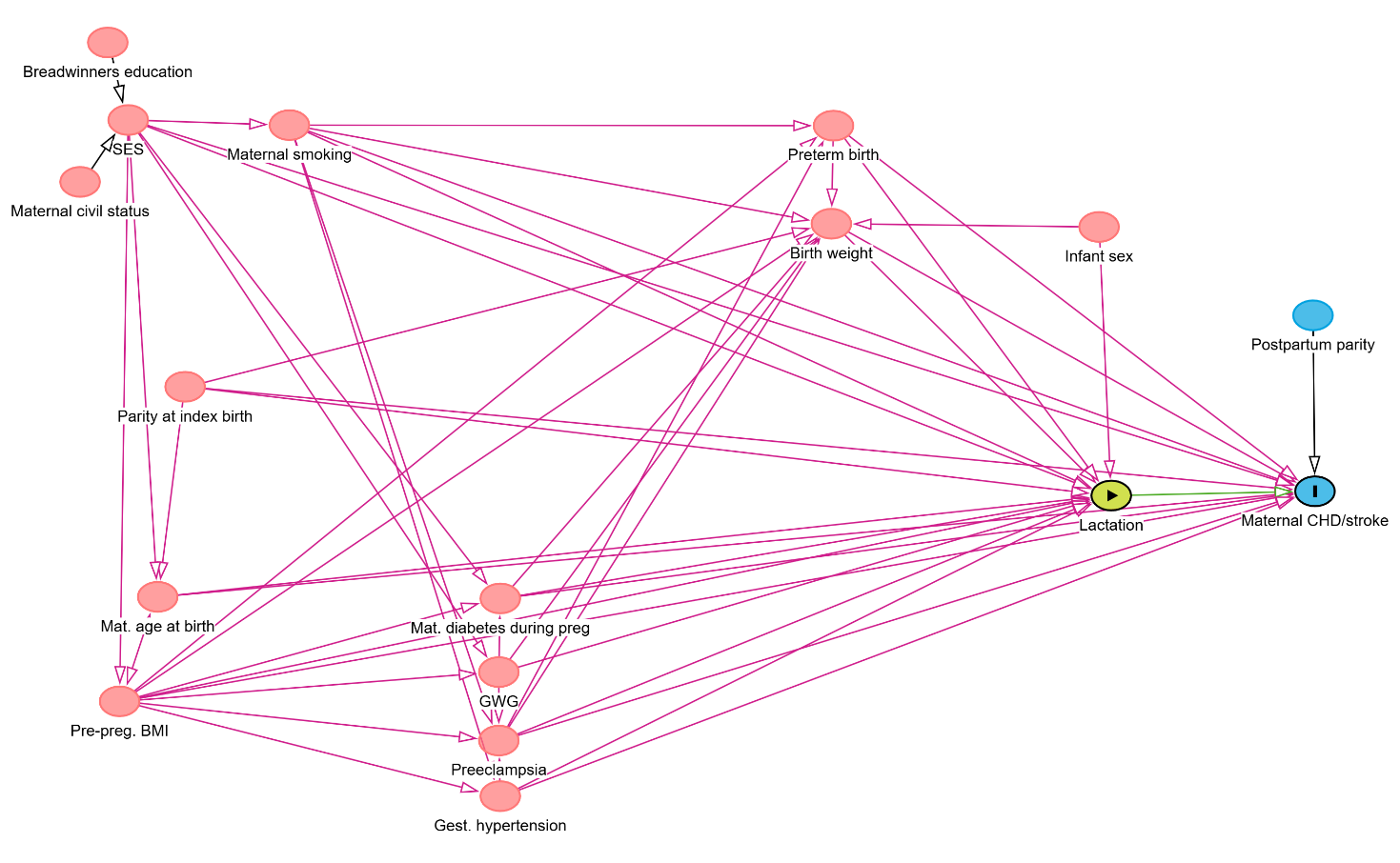


**Supplementary Figure 2.** Conceptual framework, adjusted. Adjusted for socioeconomic position, maternal age, pre-pregnancy body mass index (BMI), maternal smoking and diabetes during pregnancy, preeclampsia, gestational hypertension, parity, gestational age, and birth weight. CHD, coronary heart disease; Gest, Gestional; GWG, Gestational weight gain; Mat, Maternal; Pre-pregBMI, pre-pregnancy body mass index; SES: Socioeconomic position.


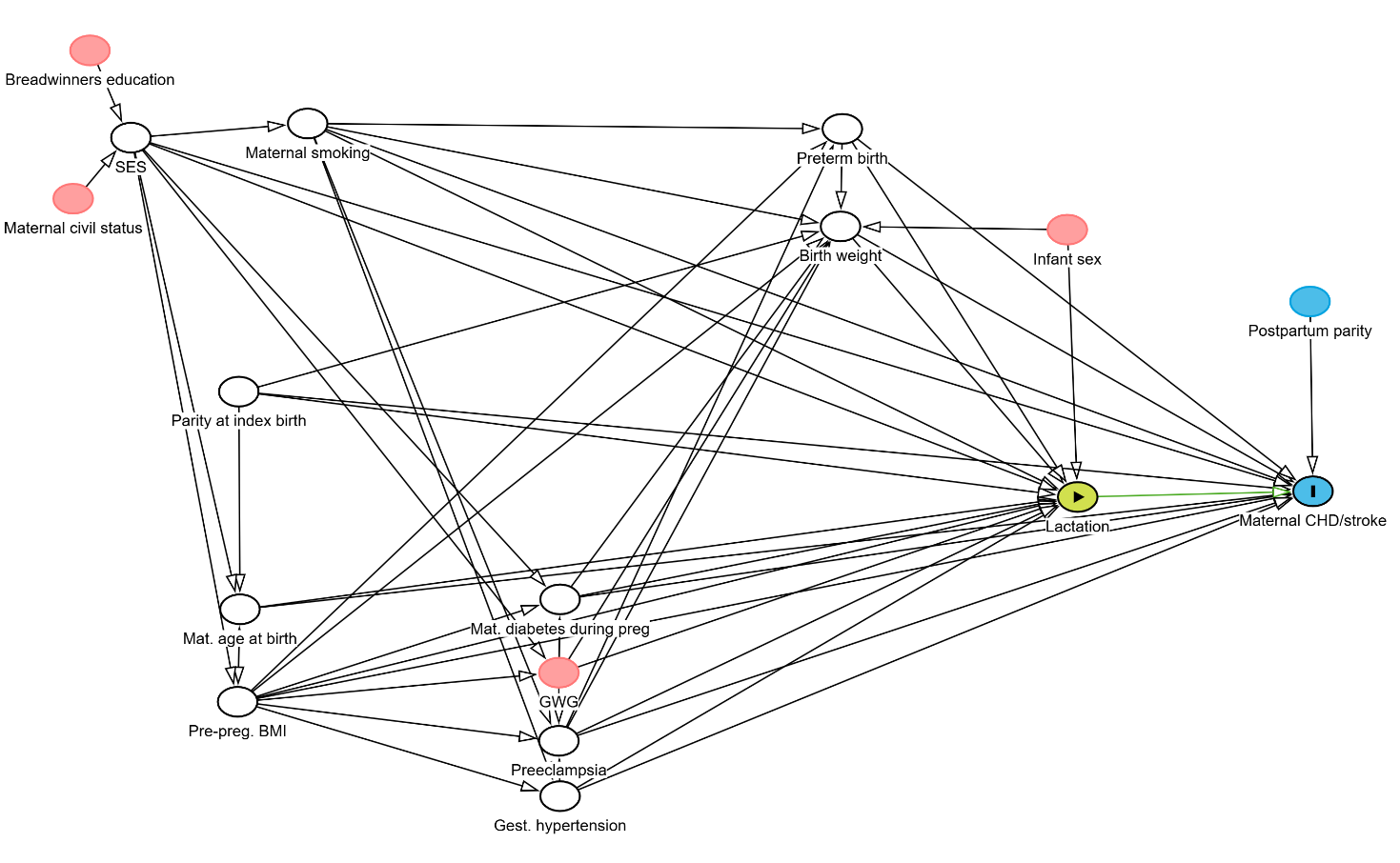


**Supplementary Figure 3.** Flow chart of the fathers.

**
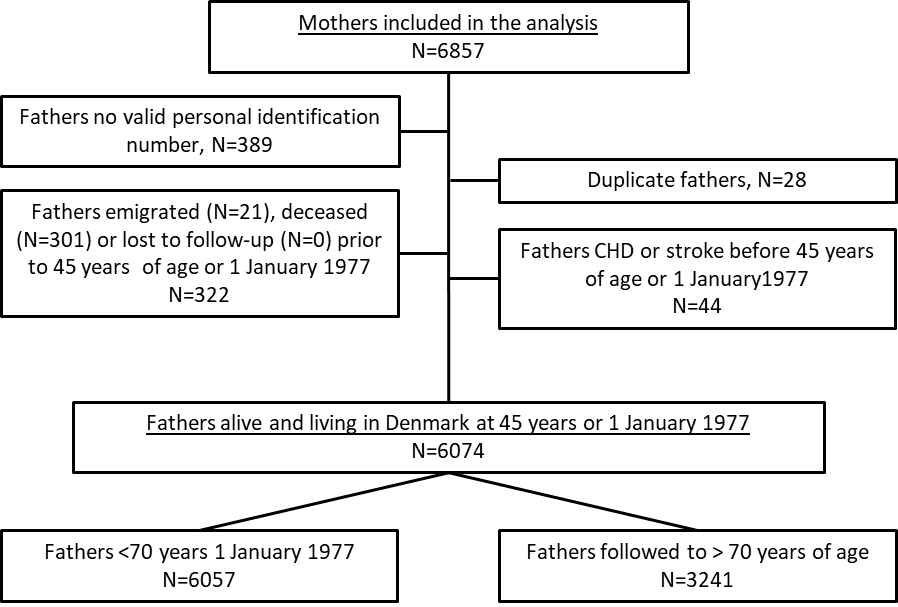
**

**Supplementary Figure 4. Associations between the duration of any lactation and risk of coronary heart disease (Panel A) and stroke (Panel B) at > 70 years of age.** The estimates are adjusted for socioeconomic position, maternal age, pre-pregnancy body mass index (PPBMI), maternal smoking and diabetes during pregnancy, preeclampsia, gestational hypertension, parity, gestational age, and birth weight.

Panel A) Panel B)


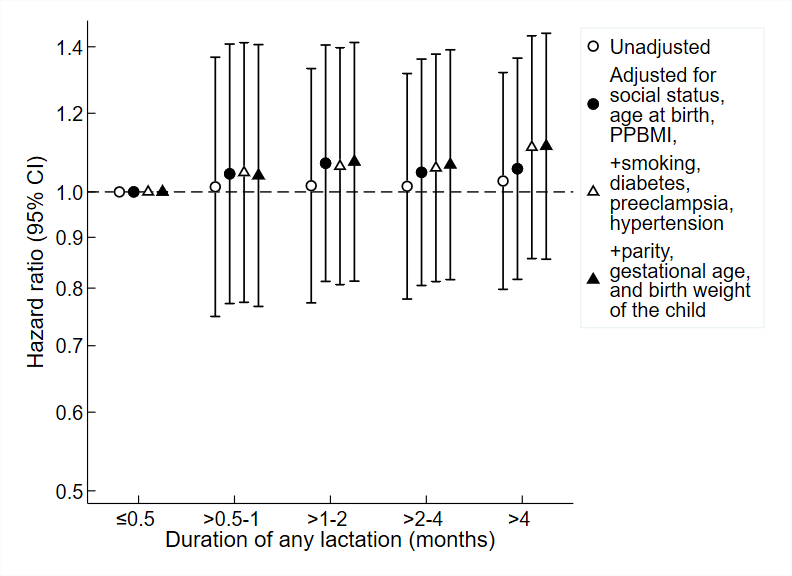

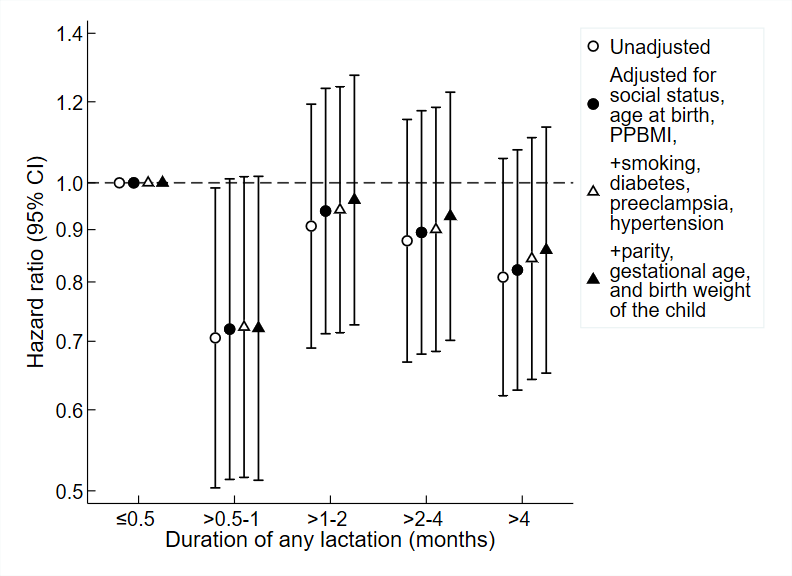

Supplement: multimedia component 1 [file mmc1.docx]
